# Video-Assisted vs. Robotic-Assisted Thoracoscopic Surgery in Lung Cancer: A Comprehensive Review of Techniques and Outcomes

**DOI:** 10.3390/jcm14051598

**Published:** 2025-02-26

**Authors:** Dina Sbeih, Mayar Idkedek, Firas Abu Akar

**Affiliations:** 1 Faculty of Medicine, Al-Quds University, East Jerusalem 20002, Palestine; dina.sbeih@students.alquds.edu (D.S.); mayar.idkedek@gmail.com (M.I.); 2 Department of General Surgery, Faculty of Medicine, Al-Quds University, East Jerusalem 20002, Palestine; 3 Department of Thoracic Surgery, The Edith Wolfson Medical Center, Holon 58100, Israel; 4 Sackler Faculty of Medicine, Tel Aviv University, Tel Aviv 6997801, Israel

**Keywords:** lung cancer, minimally invasive surgery, robotic-assisted thoracoscopic surgery (RATS), video-assisted thoracoscopic surgery (VATS)

## Abstract

Lung cancer is the primary cause of cancer-related mortality globally; hence, several medical and surgical approaches have been developed for its management. This can be easily recognized with the evolution from the traditional open thoracotomy toward minimally invasive procedures—in particular, video-assisted thoracoscopic surgery (VATS) and robotic-assisted thoracoscopic surgery (RATS)—in treating lung cancer. There has been a lot of controversy around the advantages and limitations of these procedures. VATS has been proven to be beneficial in treating early-stage lung cancer. Yet, the restricted mobility of its instruments, as well as the lack of a three-dimensional visualization of anatomical components, make the new RATS desired. RATS uses advanced technology, which has resulted in an exceptional high-definition, three-dimensional image of the working field. This has also led to fine dissection with great precision and accuracy, better lymph node removal, reduced postoperative recovery time, and better outcomes. Compared to VATS, there is less blood loss, shorter hospital stays, and less pleural effusion drainage. Despite its higher cost due to the expensive surgical systems, training and maintenance fees, and longer operative time, RATS has started to gain more use, potentially enhancing patient outcomes as experience and technological improvements progress.

## 1. Introduction

With 2.5 million incident cases in the 2022 statistics, lung cancer ranks as the most common cancer globally. It has also been considered the leading cause of cancer-related deaths worldwide, with almost 1.8 million deaths reported, equivalent to 18.7% of all cancer deaths [1]. In 2025, the American Cancer Society estimates 226,650 new lung cancer cases in the United States, with approximately 124,730 deaths [2]. This increased incidence is mainly attributed to the rise in risk factors, especially tobacco smoking [3]. Other documented risk factors include passive smoking and environmental and occupational exposures to substances such as radon gas, asbestos, chromium, and beryllium [4]. The epidemiological significance of lung cancer has made it a must to find tools for prevention, early detection, and the best possible treatment options. With the rise of technology, the treatment has shifted from the traditional thoracotomy to preferring less invasive approaches, most importantly Video-Assisted Thoracoscopic Surgery (VATS) and Robotic-Assisted Thoracoscopic Surgery (RATS) [5].

Surgery has become vital in the treatment of lung cancer, particularly for stage I and II non-small-cell lung cancer (NSCLC). In 2024, around 20.7% of lung cancer patients in the U.S. received surgical intervention [6]. Therefore, enhancements in surgical techniques and equipment became necessary. Over the years, significant advancements have undoubtedly taken place [7,8]. In previous surgeries, pneumonectomy was initially used, but afterward, the practice shifted to lobectomy and eventually to sublobar resection [9]. Furthermore, the surgical approach to the lung has evolved. Initially, it involved rib-spreading thoracotomy, using retractors to expose the lung [9]. However, this conventional method resulted in a high incidence of morbidity, particularly neuralgic pain from intercostal nerve irritation, affecting 44% of patients and leading to less favorable outcomes. Current non-rib-spreading thoracic procedures, such as VATS and RATS, are considered safer, more feasible, and have much better outcomes [9].

This review assesses the advantages and limitations of VATS and RATS and compares them in different aspects. Aiming to give physicians a better understanding of the two techniques will help them choose the best approach for each patient and suggest areas for improvement toward more patient-oriented treatment.

## 2. Video-Assisted Thoracoscopic Surgery (VATS)

### 2.1. VATS from Inception to Modern Practice

Thoracoscopy was first introduced in 1910 by the Swedish physician Hans Christian Jacobaeus, who was the first to use a uniportal technique to enter the pleural cavity [10]. Initially, thoracoscopy was mainly used for minor and diagnostic procedures, such as lysing adhesions in tuberculosis patients and performing biopsies [11]. Since then, it has evolved significantly, especially with the development of thoracoscopic cameras and the availability of endoscopic linear mechanical staplers [12,13]. These advancements led to the first VATS lobectomy in 1991 by Giancarlo Roviaro [14]. Later, in the early 2000s, thoracic surgeon Rocco was one of the first surgeons to use the uniportal approach for small operations, including wedge resections and curing pneumothorax [12]. VATS usage has not been limited to minor procedures, and over the years it has seen many changes, whether in the number of ports or the approach [15].

Learning VATS is now a must for future thoracic surgeons since it entered training programs and became part of the qualification requirements in the American Board of Thoracic Surgery [16]. VATS was and still is in the spotlight of research, and several trials have emerged, including the VIOLET prospective randomized trial, which involved 503 patients, 247 assigned to the VATS group and 256 to the open lobectomy group, with its main objective being to assess physical function. VATS resulted in significantly enhanced physical functioning after five weeks. Another outcome of this study was less incision-related pain, less analgesic consumption, and fewer major adverse events following discharge with VATS [16]. Another randomized controlled trial evaluated postoperative pain, enrolling 102 patients in the VATS group and 99 in the thoracotomy group [17]. The findings revealed a significantly lower proportion of patients experiencing clinically relevant pain within the first 24 h after VATS compared to anterolateral thoracotomy. Additionally, self-reported quality of life was significantly better after VATS, as assessed by the EuroQol 5 Dimensions (EQ5D) Questionnaire. Other notable differences were observed in terms of time: the hospital stay (median 4 vs. 5 days), epidural analgesia duration (median 2 vs. 3 days), and chest tube duration (median 2 vs. 3 days) were shorter in the VATS group. However, the thoracotomy group had a shorter surgical duration, with a median of 79 min compared to 100 min in the VATS group. Additionally, perioperative blood loss was significantly reduced in the VATS group, with a median of 50 mL, contrary to 100 mL in the thoracotomy group [17].

### 2.2. Uniportal VATS vs. Multiportal VATS

Initially, multiports up to four were used with incisions of approximately 0.5 to 1 cm for the insertion of surgical instruments, and an additional 3 to 4 cm port incision was made for tissue removal [18]. However, postoperative chest wall paresthesia related to port sites was reported by more than 50% of patients [19]. Therefore, surgeons tried to use fewer ports, and in 2011, Gonzalez-Rivas et al. reported the first uniportal VATS lobectomy [20]. In uniportal VATS, thoracoscopic access is obtained through a single 3 to 4 cm incision [18]. According to a meta-analysis [21], uniportal VATS resulted in a significantly shorter postoperative drainage time compared to two-port and multiport VATS (*p* = 0.03, *p* < 0.001, respectively), in addition to considerably shorter hospital stays, operation times, and less blood loss compared to multiport VATS (*p* < 0.001, *p* = 0.04, *p* = 0.002) but not when compared to two-port VATS. However, there was no significant difference between uniportal and multiport VATS regarding lymph node resection, conversion rate, mortality, and staging [21]. After demonstrating its superiority over multiportal VATS in most aspects, specialized uniportal VATS instruments entered the market, including the Scanlan^®^ VATS instruments. They are distinguished by a thinner and longer shaft, as well as multiple articulation points both within and outside the chest [18]. Even the noted setbacks, such as delayed poor healing caused by repeated skin extrusion, may be overcome. As mentioned by Pan et al., soft tissues and surrounding structures can be protected using wound protectors. Additionally, rather than the conventional discontinuous vertical mattress suture, a modified suturing approach for uniportal VATS is employed. This technique utilizes a single-head barbed absorbable suture and has been shown to improve wound healing, significantly reduce surgical wound infections, and enhance 30-day scar aesthetics [22].

To facilitate learning uniportal VATS, courses led by experienced surgeons are available, along with VATS simulators such as the Ethicon Stupnik VATS Simulator. Training can also include dry and animal wet lab sessions, along with visits to ultra-high-volume centers [23,24]. The initial learning period normally varies between 14 and 60 surgeries, depending on the surgeon’s previous expertise [25]. Interestingly, the learning curve for junior thoracic surgeons is shorter than for senior surgeons [26].

### 2.3. The Surgical Approach

Additionally, the approach has also seen many changes; the intercostal approach was initially used and required two or more intercostal incisions to be used for lung resection. However, this small incision in the intercostal space carries a risk of damaging the intercostal nerve, which may result in intercostal neuralgia and chronic post-thoracotomy pain syndrome [27]. Therefore, a new approach called the subxiphoid approach VATS has been developed; here, a subxiphoid incision is made along the midline of the sternum, giving surgeons access to both sides of the chest cavity and even conducting bilateral lung resections using a single incision [28]. The subxiphoid approach VATS is linked to less postoperative pain and reduced chronic postoperative paresthesia compared to the intercostal approach [27]. Since 2014, this approach has been used [29].

## 3. Robotic-Assisted Thoracoscopic Surgery (RATS)

In RATS, a three-dimensional high-definition camera and small-tipped articulating instruments, which can be inserted through 8 mm ports, are utilized. The surgeon operates these instruments from a remote control station [30].

### 3.1. The Evolution of Robotic Surgical Systems

The Da Vinci surgical system, commonly used in thoracic surgery, has evolved through four main generations: Classic (2000), S (2006), Si (2009), and Xi (2014). The system consists of a surgeon’s console, a robotic arm unit (with three standard arms and an optional fourth), and a 3D vision system. Later models include the cost-effective X-type RAS (2017) for non-oncology surgeries, the single-port RAS (2018), and the under-development Xi+. Other robotic-assisted surgical systems have also emerged, with the latest being the ’Surui’ surgical robot, approved in February 2024 [31].

### 3.2. Overview of RATS

In RATS, anatomical thoracic resections are performed by making small non-rib-spreading incisions, often utilizing 12 mm ports for the camera or stapler and 8 mm ports for robotic tools [32]. RATS offers an exceptional high-definition, three-dimensional view of the surgical field, allowing for precise and accurate fine dissection [30].

It also eliminates hand tremors and provides a clear visualization of the intricate anatomy surrounding the mediastinum and hilum. As a result, surgeons can better manage bleeding in small blood vessels [33]. The duration of postoperative chest tube drainage for RATS patients is shorter [34], consequently facilitating a quicker recovery for patients and a shorter hospital stay [34]. Nasir et al. reported that RATS enables excellent lymph node removal, a median blood loss of 20 mL, and a median hospital stay of two days, with a 10.4% conversion rate to open thoracotomy (41 of 394 cases), and the 30-day and 90-day operative mortality rates were 0.25% and 0.5%, respectively [35].

### 3.3. Cost Considerations in RATS

The primary argument against RATS is its higher cost. In a comparison study, Swanson calculated the overall cost of RATS to be $25,040.70, in contrast to $20,476.90 for VATS [36]. The cost of the robotic system itself ranged from $1 to $2 million, with an annual maintenance cost of $140,000 per system. Additionally, if an extra console for residents and fellows was purchased, as is common in teaching institutions, this would cost an extra $450,000 [35,37]. Another crucial element that played a role in the high cost of RATS is the operating room time [35]. In a retrospective study from the perspective of a Chinese healthcare payer, the 5-year costs and quality-adjusted life years (QALYs) were assessed for RATS, open thoracotomy (OT), and VATS patients. The expenditures comprised the in-hospital cost of the initial operation; the costs of individual problems, adjuvant chemotherapy, and follow-ups; and costs associated with advanced stages. Within that 5-year timeframe, patients undergoing VATS spent $18,891.88 and gained 3.58 QALYs. In contrast, RATS patients gained an additional 0.05 QALYs at an additional cost of $4006.86, resulting in an ICER of $80,324.98 per QALY compared to VATS. Therefore, while RATS was found to be cost-effective compared to OT, it was less cost-effective than VATS procedures [37]. According to Deen et al., for RATS to match the cost of VATS, the operating room time for RATS would need to be reduced to 68 min, and the cost of robotic equipment and supplies would need to be reduced to $1601 to be comparable to VATS [38]. There was a considerable variation in operating room time dependent on the surgeon’s expertise. Kaur et al. found a time difference of 71 min between the first 20 RATS operations performed by surgeons and the subsequent 22 instances, resulting in an approximate cost reduction of $883.38 in the latter surgeries [35]. Moreover, Le Gac et al. examined the learning curve (LC) associated with RATS segmentectomy and its impact on hospital-related costs, determining that proficiency was achieved after approximately 30 procedures. When comparing the final 30 procedures to the initial 30, the overall costs did not show a significant reduction in the primary analysis. However, after excluding one outlier (hospitalization-related costs > €10,000), hospitalization costs decreased by €591 (95% CI: −€1194 to −€38, *p* = 0.03), operating costs reduced by €680 (95% CI: −€1832 to +€438, *p* = 0.12), and EndoWrist™ instrument expenses declined by €135 (95% CI: −€220 to −€35, *p* = 0.004) [39]. Therefore, even if RATS is not cost-effective compared to VATS, it can still be considered cost-competitive. As surgeons and trainees become more experienced with RATS and perform more operations, a decline in operating room time and, consequently, a cost reduction can be anticipated. Furthermore, introducing additional robotic systems could drive further competitive pricing in the market [37].

## 4. VATS Compared to RATS

Nowadays, thoracic surgeons must have a solid understanding of both VATS and RATS and consider all relevant factors before determining which approach is best suited for the patient’s specific needs. Below are some of the key differences highlighted in the literature.

### 4.1. Pleural Effusion

According to a randomized controlled trial including patients suitable for minimally invasive lobectomy, 25 patients in the RATS group were compared to 50 patients in the VATS group [40]. The main results showed a statistically significant difference in the pleural effusion drained on the first and second postoperative days, with a mean pleural effusion of 140 mL in the RATS group and 214 mL in the VATS group on the first postoperative day. This may be due to RATS providing greater viewing angles, resulting in less trauma to the endothoracic tissues, reduced inflammation, and, consequently, less pleural fluid accumulation [40].

### 4.2. Postoperative Pain

The RATS group experienced significantly less postoperative pain on the first day, as measured by the Visual Analog Scale (VAS) (0.92 vs. 1.17). However, on the second postoperative day, the difference was not statistically significant [40]. Postoperative pain assessment using the Numeric Rating Scale (NRS) and painDETECT consistently showed higher scores in the RATS group compared to the VATS group at all time points measured. On postoperative days 0, 30, and 90, the NRS scores for the RATS group were 2.1, 2.0, and 0.8, respectively, while the VATS group recorded 1.6, 1.1, and 0.4, with statistically significant differences (*p* < 0.01). Likewise, painDETECT scores were higher in the RATS group at each time point (6.8, 7.4, and 4.6) compared to the VATS group (5.4, 4.5, and 2.6), also reaching statistical significance (*p* < 0.01). Further analysis of factors contributing to higher postoperative pain, such as the number of injured intercostal sites and drain tube placement, showed no significant differences. However, tumor location, the number of inserted ports, the size of the largest wound, and the use of epidural anesthesia or continuous nerve block were identified as significant risk factors for an NRS > 3 on postoperative day 30. Additionally, tumors located in the upper lobe were linked to increased postoperative pain, likely due to the wider trocar angles causing greater intercostal nerve damage [41].

### 4.3. Lymph Node Dissection

Other noted limitations of VATS, as reported in a meta-analysis, include the limited two-dimensional view and limited flexibility of the instruments, which have led to increased difficulty in performing precise lymph node dissection for staging and oncological purposes, as well as in controlling intraoperative bleeding [8]. Building on these challenges, the ROMAN study was the first randomized trial to demonstrate the advantage of RATS over VATS in terms of the number of harvested hilar lymph nodes and nodal stations. The results demonstrated a significantly higher lymph node yield with the robotic approach, with a median of seven hilar lymph nodes compared to four in VATS and seven mediastinal nodes versus five in VATS (*p* = 0.0003 and *p* = 0.0002, respectively).

### 4.4. Time

Time is a critical factor that significantly influences RATS; as the operative time is longer than VATS, the average surgery time was lower in the VATS group, being 160 min in VATS compared to 180 min in the RATS [40]. This is primarily because RATS must be performed with maximum caution, and the setup and exchange of the robot arms and forceps require additional time. Also, since RATS is relatively newer than VATS, different experiences of the surgeons with the RATS can also be a factor [40,42]. However, with increased experience using RATS, the operative time is expected to become comparable to that of VATS. This is supported by Merritt et al. reporting that the mean operative time for surgeons in their final 20 RATS procedures was significantly shorter than in the first 20 RATS procedures [43].

### 4.5. The Learning Curve

The learning curve also differs when comparing RATS to VATS. According to a retrospective study that included several certified thoracic surgeons, an estimated 28 cases were required for RATS lobectomy, while the number of cases needed for VATS was estimated to be 35 cases. The main reasons behind these differences are that most surgeons had prior VATS experience before performing RATS, as well as previous practice with the Da Vinci system and practice tests before obtaining their Da Vinci system license [44]. Further contrast is the absence of tactile feedback in RATS, which traditionally allowed surgeons to sense the resistance of tissues during procedures, a function usually achieved through palpation. This ability, during thoracoscopic dissection, is important for blunt probing and accessing planes around sensitive structures, such as veins or arteries. This is lost in RATS, as the surgeon operates from the console, positioned away from the patient. Therefore, there is no direct tactile feedback, and the surgeon has to depend solely on visual indicators for feedback [45].

## 5. Oncological Outcomes and Recurrence Following Surgical Resection

With the earlier detection of lung cancer, more cases are being diagnosed at an early stage, consequently increasing the likelihood of considering surgical procedures as a treatment option for early-stage NSCLC [46]. The most commonly performed surgical resections include lobectomy and the less extensive sublobar resection, which consists of wedge resection and segmentectomy [47]. Lobectomy remains the primary treatment for NSCLC, but segmentectomy is gaining recognition as a viable alternative for early-stage lung cancer, particularly in patients with compromised lung function. Recent trials, such as the CALGB 140503 [48] and JCOG 0802 [49] studies, have demonstrated that sublobar resections are comparable to lobectomy for small, peripheral, stage I non-metastatic NSCLC (<2 cm), establishing sublobar resections as an emerging standard of care. As the indications for sublobar resections continue to expand, determining the most appropriate surgical approach for each case becomes increasingly important.

### 5.1. Lobectomy Compared to Sublobar Resection

For years, lobectomy has been considered the gold standard for stage I NSCLC, particularly after randomized controlled trials, such as the one conducted by the Lung Cancer Study Group, reported better long-term survival and lower recurrence rates in patients with T1N0 NSCLC who underwent lobectomy as opposed to those who had sublobar resections [50,51]. However, with improved patient selection strategies, a better understanding of histological prognostic factors, and the introduction of newer diagnostic tools, there has been a resurgence of interest in sublobar resection [52]. This renewed interest is largely due to its anatomical parenchyma-sparing benefits, which not only preserve lung function but also facilitate easier future resections in case of subsequent tumor development, making it especially valuable in elderly patients where lung function preservation is crucial [53,54]. A recent meta-analysis demonstrated that the 5-year survival and recurrence rates were similar between segmentectomy and lobectomy [55]. Similar findings were reported in other studies, indicating that both resection types had similar survival rates and postoperative complications in patients with NSCLC tumors less than 2 cm in size [56,57,58]. Another important aspect to consider is postoperative lung function. According to Tane et al. [59], patients who underwent segmentectomy retained about 91.9% of their preoperative lung function, compared to 81.7% in the lobectomy group, suggesting better long-term respiratory outcomes. Moreover, the often-noted higher recurrence rate in sublobar resection, especially evident in wedge resection, compared to lobectomy may be attributed to the lack of comprehensive lymph node dissection. However, as indicated by both Brunelli et al. and Saji et al., using frozen-section analysis on segmental lymph nodes during segmentectomy can improve the comprehensiveness of nodal evaluation [49,60]. Therefore, segmentectomy can be considered a substitution for lobectomy, particularly when treating patients with comorbidities or restricted lung function, which may make lobectomy a more challenging option [55]. Nevertheless, if patients can tolerate both procedures, segmentectomy might serve as an option, particularly in cases where the tumor is a ground-glass opacity or adenocarcinoma less than 2 cm in size and located in a favorable histopathological segment [61].

### 5.2. Segmentectomy Compared to Wedge Resection

Compared to segmentectomy, which is an anatomical resection requiring the careful identification of bronchi and segmental pulmonary vessels, along with the meticulous dissection and assessment of intraparenchymal and hilar lymph nodes, wedge resection is a less demanding, non-anatomical procedure. It involves removing the malignancy along with a margin of normal lung tissue and is typically quicker, taking around 30 min, as it does not require the confirmation of bronchi and pulmonary vessels [62]. Therefore, wedge resection can be utilized for both the treatment and diagnosis of slowly progressive peripheral lesions with low PET scan uptake and is often preferred for patients with multiple comorbidities or advanced age [63]. However, according to a meta-analysis, segmentectomy resulted in significantly better overall survival, cancer-specific survival, and disease-free survival compared to wedge resection [62].

### 5.3. RATS and VATS in Lobectomy Resections

In comparing VATS and RATS lobectomy, Catelli et al. observed a significant decrease in postoperative pain on the first day, with RATS showing better results on the VAS score. On day two, although the difference was not statistically significant, 92% of RATS patients reported a VAS score of zero, suggesting that RATS may facilitate earlier mobilization and reduce the risk of lung infections. While there was no significant difference in prolonged air leaks between the groups, atrial fibrillation occurred in several VATS patients but did not occur in the RATS group. These complications were linked to using Harmonic Ace and excluding CO_2_ in the RATS procedure [40]. Overall survival and disease-free survival rates were similar between RATS and VATS, with a median follow-up of 51.7 months [64]. Additionally, no significant differences were found between the two groups regarding 5-year recurrence-free survival (RFS), overall survival (OS), or patterns of recurrence and mortality [65].

### 5.4. RATS and VATS in Sublobar Resections

A comparison of RATS and VATS was conducted in octogenarians with clinical stage IA NSCLC (tumor size ≤ 2 cm) who were undergoing minimally invasive wedge resection or segmentectomy [66]. The long-term outcomes revealed that both RATS and VATS sub-lobectomy exhibited similar 5-year overall survival (OS) and recurrence-free survival (RFS), consistent with previous studies [67,68] despite differences in patient age groups, suggesting that both techniques offer similar oncologic efficacy for octogenarians with early-stage NSCLC. Overall, RATS may offer advantages such as a reduced hospital stay and improved lymph node assessment while maintaining equivalent long-term outcomes to VATS in sub-lobar resections for early-stage NSCLC.

## 6. Discussion

Surgical resection takes up a large part of the treatment plan for lung cancer. For decades, surgeons used the traditional invasive thoracotomy, especially in treating NSCLC. However, the surgical route is currently shifting toward minimally invasive procedures, such as VATS and RATS [69]. VATS overcomes thoracotomy in the aspect of having better physical functioning at five weeks, less analgesic consumption, fewer serious adverse events after discharge, reduced postoperative pain, improved quality of life, shorter surgical durations, less perioperative blood loss, and a shorter hospital stay [17,70]. Additionally, uniportal VATS is now gaining superiority over multiport VATS, with some surgeons finding it easier to learn due to the alignment of the hands and eyes in the same field [18]. One study has shown that junior thoracic surgeons experience a shorter learning curve compared to senior surgeons [71]. With the rise of technology, evolution has occurred in minimally invasive procedures, leading to the appearance of RATS. RATS started gaining more popularity due to its three-dimensional view of the operating field and the greater range of motion, which is not found in VATS; this has led to increased accuracy and precision in dissection [72,73]. RATS also has shorter hospital stays, less conversion to thoracotomy, less postoperative pain, and less postoperative pleural effusion drainage [33,35,40]. While some studies [74,75] suggest that RATS has a longer operative time, a meta-analysis [76] and several other studies [8,77] found no significant difference between RATS and VATS. An additional study [43] attributed the longer duration to the initial learning curve, as surgeons have less experience with RATS. However, operative time is expected to decrease as proficiency with the technique improves. As for intraoperative blood loss, it was significantly lower in RATS when compared with VATS [76,78], likely due to the enhanced three-dimensional magnified view and more flexible instruments, which provide better visualization of the complex anatomy around the hilum and mediastinum, allowing for improved bleeding control [79]. The superior stability and enhanced vision in RATS have also enabled surgeons to conduct an extensive lymphadenectomy and contributed to an improved lymphadenectomy, particularly with a significantly greater number of dissected lymph node stations [76,78,80,81,82,83]. Additionally, RATS patients benefit from shorter drainage times and hospital stays, attributed to the more delicate approach, reduced tissue irritation, and enhanced hemostasis. These factors minimize inflammation and pleural effusion, ultimately leading to a faster recovery and reduced postoperative duration [78].

Some studies [77,84] have reported a lower mortality rate in RATS compared to VATS, particularly in the 30-day postoperative period. Additionally, RATS has been linked with a lower rate of postoperative complications [8]. The 3-year overall survival (OS) in RATS was not inferior to that in VATS, indicating comparable long-term outcomes between the two techniques [42]. Postoperative pain assessment using the Numeric Rating Scale (NRS) and painDETECT [41] showed consistently higher scores in the RATS group compared to the VATS group. This difference was attributed to several risk factors, including the number of inserted ports and the administration of epidural anesthesia or continuous nerve block.

Furthermore, tumors located in the upper lobe were linked to higher postoperative pain, likely due to the wider trocar angles, which resulted in more damage to the intercostal nerves. These findings could offer important guidance for optimizing port placement in future procedures [41]. Other postoperative complications occurred in 32% of the RATS group, compared to 46% in the VATS group; the most common complications were prolonged air leaks from the chest drain and the development of atrial fibrillation and other cardiac arrhythmias [41]. The cost of RATS is what creates controversy when comparing it to VATS due to its higher price, which could make physicians hesitate to choose it despite all its mentioned benefits [36]. The higher cost of RATS arises not only from the robotic system itself but also from additional expenses such as maintenance, training programs, and disposable tools. However, there is optimism that these costs will decrease in the future, especially with more companies like Medtronic and the collaboration between Johnson & Johnson and Google in developing new robots [85,86,87]. This innovation may help reduce costs and improve the widespread use of RATS. As the technique becomes more widely adopted and surgeons become more proficient, we can expect improvements in outcomes such as shorter operative times, reduced hospital stays, and ultimately lower overall costs [88]. The debate extends beyond RATS versus VATS, as the optimal surgical approach (segmentectomy versus lobectomy) remains a controversial topic for thoracic surgeons. A post hoc analysis of the JCOG0802/WJOG4607L trial [89] explored the outcomes of segmentectomy versus lobectomy for stage IA NSCLC. Segmentectomy was associated with better 5-year overall survival (OS) in patients aged 70+ and males, while lobectomy showed improved 5-year relapse-free survival (RFS) in younger patients and females. These findings suggest segmentectomy may benefit older or male patients with pure-solid NSCLC, but outcomes depend on age and sex. Despite segmentectomy’s potential OS advantage, it carries a risk of higher local recurrences, which may explain the historical preference for lobectomy. Recent reviews support this, but factors such as lymphadenectomy extent, tumor characteristics, and patient selection influence outcomes. Additional high-quality studies are necessary to improve patient selection and identify the most effective surgical approach for stage I NSCLC [89,90]. When comparing RATS and VATS for sublobar and lobectomy resections, both techniques demonstrate similar long-term outcomes in terms of survival and recurrence rates. RATS lobectomy showed less postoperative pain than VATS on day one [40]. Overall survival, disease-free survival, and 5-year recurrence-free survival rates were similar between RATS and VATS lobectomy, with no significant differences in patterns of recurrence or mortality [64,65]. The prevalence of NSCLC is increasing in octogenarians, a group that faces higher perioperative mortality due to comorbidities and reduced cardiopulmonary function. As a result, selecting the optimal surgical approach for sublobar resection in this age group is essential. When comparing RATS and VATS in octogenarians with clinical stage IA NSCLC (tumor size ≤ 2 cm) undergoing minimally invasive wedge resection or segmentectomy, the RATS approach led to a shorter postoperative hospital stay and a statistically significant reduction in intraoperative bleeding, although the difference was clinically insignificant, and the long-term outcomes showed that both approaches provided comparable 5-year OS and RFS in sublobar procedures [66]. A limitation of our review is that most of the studies included were conducted in high-income countries, with insufficient data available from low-income countries. Notably, India has only recently begun to implement robotic surgery. The first experiences in robotic surgery for lung diseases include decortication, segmentectomy, and pneumonectomy [91]. Therefore, we recommend further studies in low-income countries to evaluate the adoption and outcomes of RATS, assess knowledge and training levels, and understand the challenges related to healthcare infrastructure and access to advanced technologies in these regions.

## 7. Conclusions

RATS represents a significant breakthrough in thoracic surgery, offering numerous advantages that often outweigh its disadvantages. While it may be more expensive, its postoperative outcomes can justify the higher costs. As surgeons gain more experience and the number of RATS procedures increases, expanded insurance coverage and market competition could lead to more reasonable costs. This review emphasizes the importance of tailoring surgical options to each patient, considering factors such as age, health status, and long-term quality of life. We recommend further research comparing RATS and VATS in both lobar and sublobar resections, taking into account diverse surgical experiences, varying levels of expertise, and the learning curve among thoracic surgeons. Additionally, larger studies should assess patient selection, healthcare system differences, and surgeon experience for a more comprehensive evaluation of RATS. Optimizing RATS outcomes requires comprehensive training programs and standardized protocols to enhance safety and effectiveness. The learning curve could be improved by training in high-volume centers, attending courses and conferences, watching surgical videos, and participating in wetlabs and drylabs.

## Data Availability

No new data were created or analyzed in this study.
